# Knowledge of heart attack and stroke symptoms among US Native American Adults: a cross-sectional population-based study analyzing a multi-year BRFSS database

**DOI:** 10.1186/s12889-020-8150-x

**Published:** 2020-01-10

**Authors:** Jared C. Van Hooser, Krista L. Rouse, Mandy L. Meyer, Amanda M. Siegler, Beth M. Fruehauf, Elliot H. Ballance, Sarah M. Solberg, Michael J. Dibble, M. Nawal Lutfiyya

**Affiliations:** 1 University of Minnesota, College of Pharmacy, Duluth, MN 55812 USA; 2 Essentia Health System, Duluth, MN 55805 USA; 3 Oakeaf Surgical Hospital, Altoona, WI 54720 USA; 4 Security Health Plan, Marshfield, WI 54449 USA; 5 Lutheran Hospital, Fort Wayne, IN 46804 USA

**Keywords:** Heart attack symptoms, Stroke symptoms, Knowledge of heart attack and stroke symptomology, Native American adults

## Abstract

**Background:**

Heart disease and stroke are among the leading causes of death in Native Americans. Knowledge of heart attack and stroke symptomology are essential for prompt identification of symptoms and for appropriate action in seeking care. Knowledge of heart attack and stroke symptoms among US Native American adults was this study’s focus.

**Methods:**

Multivariate techniques were used to analyze national surveillance data. Native American adults comprised the study population. Low heart attack and stroke knowledge score was the dependent variable.

**Results:**

Logistic regression analysis yielded that Native American adults with low heart attack and stroke composite knowledge scores were more likely to be: older, less educated, poorer, uninsured, a rural resident, male, without a primary health care provider, and lacking a recent medical checkup.

**Conclusions:**

The identified characteristics of Native American adults with heart attack and stroke knowledge deficits or disparities should guide educational initiatives by health care providers focusing on improving such knowledge.

## Background

The disease burden of heart attacks and strokes is considerable, not only in the United States (US) but worldwide [1]. Globally, an estimated 17.9 million people died from cardiovascular disease (CVD) in 2016, representing 31% of all global deaths. Of these deaths, 85% were due to heart attacks and strokes [1]. Annually, in the US the costs of CVD and stroke is an estimated $316.1 billion [2] of which $189.7 billion are direct costs and $126.4 billion indirect costs. Direct costs include the cost of health care professionals, hospitalizations, prescription medication, and home health care (not including nursing home care), while indirect costs include lost productivity associated with premature CVD and stroke related death. Stroke and CVD comprise 14% of total health expenditures in the US, which is greater than any other major diagnostic group [2].

Native Americans, including both American Indians (AI) and Alaskan Natives (AN), are more susceptible to heart disease and stroke than non-Hispanic Caucasians [3]. At the time of the last US Census, Native Americans comprised an estimated 2.0% (6.2 million people) of the US population [4]. Native Americans are part of 569 federally recognized tribes [5]. Thirty-four percent of Native Americans live on reservations located in rural areas and another 55% live in urban communities [6]. Since 1955 Native Americans have been eligible for healthcare from the Indian Health Service (IHS) [7]. Despite being eligible for healthcare, Native Americans experience greater health disparities when compared with other US populations. For instance, compared to the average life expectancy of the general US population, Native Americans have an average life expectancy that is 5.5 years lower [8]. Also, heart disease is the first and stroke the seventh leading cause of death for Native Americans as well as causes of major morbidity [8–10].

Over the past few decades there has been a stark change in heart disease death rates among Native Americans. Although heart disease death rates for Native Americans were 21% lower than the total US population in the early 1970s, they were 20% higher by the late 1990s [6, 11]. Likewise, the incidence of stroke has increased since the 1970s. From 1972 to 1985, stroke death rates for Native Americans declined, albeit slower than the total US population [6, 11]. From 1985 to 1997 there was little to no decline in stroke death rates for Native Americans [6, 11]. However, at the end of the 1990s, stroke death rates were 14% higher for Native Americans than for the total US population [6, 11]. There is some evidence that this trend has continued [12].

Additionally, Native Americans have 2.3 greater odds of being diagnosed with diabetes than non-Hispanic Caucasians [13], a condition that is a risk factor for heart attack and stroke [14, 15]. Death from heart disease is approximately two to four times higher in adults with diabetes than adults without diabetes [16]. Also, stroke risk is two to four times higher in adults with diabetes [16]. Further, over 25% of Native Americans have high blood pressure [2], another risk factor for heart attack and stroke [9]. Finally, Native Americans die from heart disease at younger ages than other racial and ethnic groups in the US [17]. Thirty–6 % of Native Americans who die from of heart disease die before age 65, over double the rate of the total US population (17%) [17].

Knowledge is the first line of prevention for heart attack and stroke, making it essential to assess symptom knowledge for these conditions in this high-risk population. Studies have shown that there are disparities in adult awareness of heart attack warning signs and symptoms with regard to race, sex and ethnicity [18–23]. Recent research has shown that Native Americans are aware of their risk for heart disease and stroke [24, 25]. However, a small, recent study among Native Americans in the urban southwest, provided preliminary evidence suggesting that awareness of heart attack and stroke symptoms was lower than that of the total US population [26].

Heart attack and stroke are medical emergencies that need immediate recognition of symptoms and appropriate prompt decisions [27, 28]. Patients who receive treatment more quickly are more likely to avoid the disabling effects of stroke, and have decreased mortality compared to those whose treatment is delayed [29]. A recent study suggests less than 27% of stroke victims receive tissue plasminogen activator within 60 min of presentation [30]. Heart attack is primarily treated by coronary revascularization, or procedures to restore blood flow to heart muscle, via percutaneous coronary intervention (PCI) or coronary artery bypass graft (CABG) [31, 32]. It is crucial that the PCI and CABG procedures are performed in a timely fashion [31].

This study sought to investigate the knowledge levels of heart attack and stroke symptoms among Native American adults. The findings of this study could inform the development of clinical or public health interventions to ameliorate possible knowledge disparities.

## Methods

Multiple years of the heart attack and stroke module from the Behavioral Risk Factor Surveillance System (BRFSS) were studied using complex samples bivariate and multivariable techniques. BRFSS is a health related telephone survey that is overseen by the Centers for Disease Control and Prevention. It is completed is all 50 US states and several US territories, collecting information from adults > 18 years of age. After being collected, BRFSS data are weighted in order to represent the noninstitutionalized US population by surveyed state. Complex multi-stage sampling techniques are used for data collection. The BRFSS survey design, data collection, and sampling measures have been previously described [33], as well as similar methods on the BRFSS heart attack and stroke module in different populations [20–23].

The study population was Native American adults. Data from the BRFSS module on heart attack and stroke symptomology were used in these analyses. This optional module is available for data collection every other year and different states use this module in different years. In order to include as many US states and US territories as possible and to include as large a sample of Native American adults as possible, data from 2005, 2007, and 2009 was merged. Further, since BRFSS weighting methodology was amended we chose the 3 years of data to merge that we did to ensure consistency in the data weighting. Data from 25 states, the United States Virgin Islands (USVI), and the District of Columbia were used in these analyses. If a state used the heart attack and stroke symptomology module in multiple years, only the data from the most recent year were used.

The BRFSS heart and stroke symptomology module consisted of 13 questions which assess a person’s knowledge of early symptoms of heart attack and stroke. Six of the questions were on heart attack symptoms. BRFSS participants were asked if the following were symptoms of a heart attack: pain or discomfort in the jaw, neck, or back; feeling weak, lightheaded, or faint; chest pain or discomfort; pain or discomfort in the arms or shoulders; shortness of breath. An incorrect symptom, trouble seeing in one or both eyes, a symptom of a stroke, was included to examine the possibility that participants would answer “yes” to every question. Six questions were on stroke symptoms. BRFSS participants were asked if the following were symptoms of stroke: sudden confusion; trouble speaking or understanding; sudden numbness or weakness of face, arm, or leg; sudden trouble seeing in one or both eyes; sudden trouble walking, dizziness, or loss of balance or coordination; or sudden, severe headache with no known cause. In a similar fashion to the questions on heart attack symptoms, an incorrect symptom for stroke, sudden chest pain, was included to examine the possibility that participants would answer “yes” to every question. The last question asked participants what would be the first thing they would do if they thought someone was having a heart attack or a stroke: take them to the hospital, tell them to call their doctor, call 911, call their spouse or family member, or do something else [20, 34].

The questions from the heart attack and stroke symptom module were grouped together for analysis because both heart attack and stroke are vascular medical emergencies that require the immediate identification of the symptoms by patients and bystanders. The questions were also grouped together because any public health endeavors will likely need to focus both heart attack and stroke symptom knowledge together, as strokes are frequently called “brain attacks,” and many facets of initial stroke care are similar to initial heart attack care [35, 36].

For each BRFSS participant, we calculated a heart attack and stroke knowledge score where one point for assigned for each correct response. To categorize the knowledge scores, a low score was assigned 0–5 points, a midrange score 6–9 points, and high score 10–13 points. Albeit somewhat arbitrary like many scales, the cut points were based on the possible range, 0–13, from the BRFSS module. This scale and similar ones used with the heart attack and stroke module have been previously published and allow for the standardized comparison of knowledge scores [20–23].

The covariates for the analysis included: sex, age, annual median household income, education completed, marital status, geographic locale, timing of last routine medical checkup, having a personal health care provider (HCP), having health insurance, deferment of medical care because of cost, and self-defined health status. Similar covariates have been studied in heart attack and stroke composite knowledge scores [20–23].

Respondents geographic location was recoded as rural or non-rural using the Metropolitan Statistical Area (MSA) variable from BRFSS. Rural residents were defined as all participants living outside an MSA or living within an MSA that had no center city. Non-rural residents were defined as people living in a center city of an MSA, outside the center city of an MSA but inside the county containing the center city, or inside a suburban county of an MSA.

The BRFSS variables that were recoded for the analyses included: age, education completed, marital status, and annual household income. Age was collapsed and recoded as a categorical variable and split into three levels: 18–44 years, 45–64 years, and ≥ 65 years. For some variables with multiple responses options, education, marital status and household income, recoding was done to reduce the number of categories. Education completed was collapsed into less than high school, at least high school, or university graduate. Annual household income was recoded into the groups of <$50,000 and ≥ $50,000. Marital status was recoded into two groups: married or living with a partner and not married or living with a partner.

The following BRFSS variables that focus on access to health care were also included in the analyses: having a HCP, deferment of medical care because of cost, and health insurance status. Any response recorded as “don’t know” or “refused to answer” were treated as missing data and were omitted from the analyses.

The statistical analysis of national survey data such as BRFSS must reflect the survey’s complex sample design [37]. As such complex samples bivariate and multivariable analysis were performed. Saylor, et al., states, “Complex sample data analysis adjusts for weights, cluster, and stratification of the sampling design to produce unbiased national estimates of population means and frequencies from the sample after taking into account weights for over- or undersampling of specific groups. Complex sample analysis provides estimates of variability based on the number of cases in the sample rather than the number of cases in the population.” [37]

Complex bivariate analysis describing the three-level composite scores (low, midrange, and high score) by each covariate was completed. Correct and incorrect answers for each heart attack and stroke symptomology knowledge question by Native American status was also analyzed. One complex samples logistic regression model was performed using low scores, on the combined heart attack and stroke symptomology knowledge questions, as the dependent variable. All of the study covariates were entered into the model. Alpha was set at 0.05 for all tests of statistical significance. SPSS version 25.0 (IBM, Chicago, IL) was used to complete the analyses. The Institutional Review Board (IRB) at the researchers’ institutions acknowledge that the analysis of de-identified and publicly available data does not constitute human subjects research as defined in federal regulations and thus does not require IRB review. As this was a de-identified data only study, IRB approval was not required.

## Results

Table 1 describes the Native American population responding to the optional heart attack and stroke symptom module questions in 2005, 2007 and 2009 BRFSS. From the multiyear amalgamated database compiled for this study, a weighted population of 7,336,666 Native American adults ≥18 years of age from 25 states, the USVI and the District of Columbia were identified. Proportionately, this population was younger with 53.2% being 18–44 years of age. The majority reported having an annual median household income under $50,000 (68.6%). Geographically, in comparison to the overall US adult population, a higher proportion of Native American adults reported living in rural rather than non-rural areas (33.7% versus 18.0%). Almost a quarter of the respondents defined their health as fair to poor (24.2%). Over 20% of respondents reported that they had no health insurance (23.8%), no health care provider (25.8%) and deferred medical care because of cost (21.6%), all within the past 12 months. Further, 35.1% reported not having had a routine medical check-up within the past 12 months. Finally, slightly less than 50% of this population received a low, two-level composite heart attack and stroke symptom knowledge score.

**Table 1:** Description of Native American Population. Amalgamated 2005, 2007, 2009 BRFSS Database (weighted *n* = 7,336,666)

Variables and Factors	Percent
Sex	
Male	51.9
Female	48.1
Age	
18–44 Years Of Age	53.2
45–64 Years Of Age	33.8
> = 65 Years Of Age	13.0
Annual Median Household Income	
<$50,000	68.6
> = $50,000	31.4
Education Attained	
Did Not Graduate From HS	17.7
HS Graduate	61.4
University Graduate	20.9
Geographic Locale	
Non-Rural	66.3
Rural	33.7
Health Status	
Good To Excellent Health	75.8
Fair To Poor Health	24.2
Health Insurance Status	
Have Health Insurance	76.2
Do Not Have Health Insurance	23.8
Have Health Care Provider	
Have HCP	74.2
Do Not Have HCP	25.8
Medical Care Deferred	
Medical Care Deferred Because Of Cost	21.6
Medical Care Not Deferred	78.4
Routine Medical Check Up	
Within Last 12 Months	64.9
Longer Than 12 Months Ago	35.1
Three Level Composite Knowledge Score	
Low Composite Score	8.7
Mid-Range Composite Score	40.9
High Composite Score	50.4
Two Level Composite Knowledge Score	
High Score	50.4
Low Score	49.6

Table 2 displays the complex samples stratified analysis of the three-level composite heart attack and stroke knowledge scores by the study covariates or predictor variables. Row percentages by variable factors are reported. Notably this analysis yielded statistically significant results by chi square (*p* = <.05) for the variables age, education completed, household income, have personal healthcare provider, and routine medical checkup.

**Table 2:** Composite Heart Attack and Stroke Symptomology Knowledge Score By Covariates for Native American Adults Complex Samples Analysis. Amalgamated 2005, 2007 and 2009 BRFSS Data

Covariates and Factors	Three Level Composite Score		
	% Low Composite Score	% Mid-Range Composite Score	% High Composite Score
Sex			
Male	9.1	41.7	49.2
Female	8.2	40.1	51.7
Age*			
18–44 Years	8.1	44.2	47.8
45–64 Years	6.9	35.7	57.5
> = 65 Years	16.8	40.5	42.7
Education Attained*			
Did Not Graduate From HS	16.1	48.2	35.7
HS Graduate	7.9	42.7	49.3
University Graduate	4.3	29.3	66.4
Household Income*			
<$50,000	10.0	45.1	44.9
> = $50,000	3.7	30.4	65.9
Geographic Locale			
Non-Rural	8.3	40.1	51.6
Rural	9.3	42.2	48.5
Health Status			
Good To Excellent Health	8.0	41.7	50.3
Fair To Poor Health	10.8	38.2	51.0
Health Insurance Status*			
Have Health Insurance	8.1	39.2	52.8
Do Not Have Health Insurance	10.5	46.4	43.1
Have Health Care Provider*			
Have HCP	8.3	38.3	53.3
Do Not Have HCP	9.8	49.2	41.0
Medical Care Deferred			
Deferred Because Of Cost	7.8	38.3	53.9
Care Not Deferred	8.8	41.7	49.5
Routine Medical Check Up*			
Within Last 12 Months	7.7	39.0	53.3
Longer Than 12 Months Ago	10.4	44.4	45.2

*Significantly different by chi-square test of independence at the .05 level

A bivariate analysis of the percent correct and incorrect responses by symptom question and race/ethnicity status of respondents (Caucasian, African American, Native American, and Hispanic) is displayed in Table 3. For all of the questions on heart attack and stroke symptomology, Native American respondents had higher percentages of *incorrect* answers when compared to their Caucasian counterparts.

**Table 3:** Heart Attack and Stroke Symptomology Knowledge Questions With Percent Incorrect and Correct Responses for US Adults By Race/Ethnicity. Amalgamated 2005, 2007 and 2009 BRFSS Data

Survey Question (correct answer)	Race Ethnicity				Total US
	Caucasian	African American	Native American	Hispanic	
Do you think pain or discomfort in the jaw, neck, or back are symptoms of a heart attack? (Yes)					
Incorrect Answer	43.4	62.2	50.8	68.8	47.8
Correct Answer	56.6	37.8	49.2	31.2	52.2
Do you think feeling weak, lightheaded, or faint are symptoms of a heart attack? (Yes)					
Incorrect Answer	34.5	46.6	38.3	46.8	37.1
Correct Answer	65.5	53.4	61.7	53.2	62.9
Do you think chest pain or discomfort are symptoms for a heart attack? (Yes)					
Incorrect Answer	5.4	12.2	8.6	18.7	7.3
Correct Answer	94.6	87.8	91.4	81.3	92.7
Do you think sudden trouble seeing in one or both eyes is a symptom of a heart attack? (No)					
Incorrect Answer	56.6	59.5	60.9	61.6	57.4
Correct Answer	43.4	40.5	39.1	38.4	42.6
Do you think pain or discomfort in the arms or shoulders are symptoms of a heart attack? (Yes)					
Incorrect Answer	10.0	24.9	13.9	32.0	13.6
Correct Answer	90.0	75.1	86.1	68.0	86.4
Do you think shortness of breath is a symptom of a heart attack? (Yes)					
Incorrect Answer	13.0	20.3	18.5	25.8	14.9
Correct Answer	87.0	79.7	81.5	74.2	85.1
Do you think sudden confusion or trouble speaking are symptoms of a stroke? (Yes)					
Incorrect Answer	7.8	17.0	14.8	31.4	10.8
Correct Answer	92.2	83.0	85.2	68.6	89.2
Do you think sudden numbness or weakness of face, arm, or leg, especially on one side are symptoms of a stroke? (Yes)					
Incorrect Answer	4.5	9.6	7.8	19.8	6.3
Correct Answer	95.5	90.4	92.2	80.2	93.7
Do you think sudden trouble seeing in one or both eyes is a symptom of a stroke? (Yes)					
Incorrect Answer	25.9	36.7	32.2	44.0	28.7
Correct Answer	74.1	63.3	67.8	56.0	71.3
Do you think sudden chest pain or discomfort are symptoms of a stroke? (No)					
Incorrect Answer	57.3	70.1	64.8	63.9	59.6
Correct Answer	42.7	29.9	35.2	36.1	40.4
Do you think sudden trouble walking, dizziness, or loss of balance are symptoms of a stroke? (Yes)					
Incorrect Answer	12.4	20.8	19.4	29.7	14.8
Correct Answer	87.6	79.2	80.6	70.3	85.2
Do you think severe headache with no known cause is a symptom of a stroke? (Yes)					
Incorrect Answer	37.7	42.1	47.5	42.7	38.8
Correct Answer	62.3	57.9	52.5	57.3	61.2
If you thought someone was having a heart attack or a stroke, what is the first thing you would do? (call 911)					
Incorrect Answer	12.6	13.4	17.4	17.0	13.0
Correct Answer	87.4	86.6	82.6	83.0	87.0

Complex samples logistic regression analysis, displayed in Table 4, showed that Native American adults who had low composite heart attack and stroke knowledge scores had greater odds of not being a university graduate but rather a high school graduate (OR 1.578, 95% CI 1.058–2.352) or to not have graduated from high school (OR .283, 95% CI 1.443–3.643). Further, low scoring Native American adults had greater odds of being ≥65 years of age (or lower odds of being 45–64 years of age rather than 18–44 years of age). Finally, low scoring Native American adults had greater odds of living in a household with an income < $50,000 (OR 1.938, 95% CI 1.378–2.724).

**Table 4:** Complex Samples Logistic Regression Analysis of US Native American Adults with Low Heart Attack and Stroke Symptom Knowledge Score. Amalgamated 2005, 2007, 2009 BRFSS Data

Variables	Factors	Adjusted Odds Ratio	95% Confidence Interval	
			Lower	Upper
Age	45–64 Years Of Age vs. 18–44 Years Of Age	.756	.587	.973
	> = 65 Years Of Age vs. 18–44 Years Of Age	1.130	.827	1.543
Sex	Male vs. Female	1.202	.930	1.554
Education Completed	Did Not Graduate From HS vs. University Graduate	2.293	1.443	3.643
	HS Graduate vs. University Graduate	1.578	1.058	2.352
Health Status	Fair To Poor Health vs. Good To Excellent Health	.867	.666	1.128
Health Insurance Status	Do Not Have Health Insurance vs. Have Health Insurance	1.250	.882	1.771
Have Health Care Provider	Do Not Have HCP vs. Have HCP	1.150	.818	1.615
Medical Care Deferred	Medical Care Deferred Because Of Cost vs. Medical Care Not Deferred	.699	.488	1.000
Routine Medical Check Up	Longer Than 12 Months Ago vs. Within Last 12 Months	1.138	.855	1.515
Geographic Locale	Rural vs. Non-Rural	1.022	.821	1.272
Household Income	<$50,000 vs. > = $50,000	1.938	1.378	2.724

## Discussion

This study identified the characteristics of Native American adults with low knowledge of heart attack and stroke symptomology. Among the significant characteristics identified, were that this population was older, poorer, less educated, and have health service deficits (no health insurance, no personal HCP, last routine medical check-up longer than 12 months ago, and deferring medical care because of cost) [38, 39]. This study was the first that we know of to specifically examine heart attack and stroke symptomology knowledge of the Native American adult population. Multiple studies have demonstrated a strong association between lower education completion and low knowledge of heart attack and stroke symptoms in various populations [20–23, 40–43] and this was congruent with our findings.

Our results suggest that overall Native American adults have a knowledge deficit regarding heart attack and stroke symptomology compared to the total US population. Racial disparities in knowledge of heart attack and stroke symptoms have been identified for non-Caucasian populations in previous studies [20, 21, 44, 45].

Knowledge deficits regarding heart attack and stroke symptomology among the Native American population is particularly concerning for several reasons. Heart attack and stroke can lead to morbidity and are leading causes of death among Native Americans [8–10]. Furthermore, Native Americans die from heart disease at younger ages than other ethnic and racial groups in the US---over double the rate of the total US population under 65 who die from heart disease [17]. Moreover, this population is at a greater risk of being diagnosed with diabetes and hypertension, both of which are risk factors for heart attack and stroke [2, 13–20]. Prompt and proper recognition of symptoms are crucial to reducing debilitating effects and death related to heart attack and stroke [27–31].

Our findings have identified that Native American adults have health service deficits suggesting that this group has barriers to health care access. A 2006 regional study from Minnesota focusing on barriers to health care found that American Indians were more likely to report racial discrimination, cultural misunderstandings, family/work responsibilities, and transportation challenges [46]. Specific barriers to health care access varied among racial groups as non-Hispanic Caucasians were more likely to report being unable to see their preferred provider as a barrier [46]. Although this study included individuals who were enrolled in health care programs, access barriers were still encountered and significant differences were detected in challenges cited by American Indians and non-Hispanic Caucasians [46].

Despite the existence of the IHS, that theoretically provides universal health care services to Native Americans, there is a surprising number of Native Americans who report being uninsured. Previous research using BRFSS data noted that compared to non-Hispanic Caucasians, Native Americans had a lower prevalence of insurance coverage or access to a personal HCP [47]. A recent study analyzing the National Survey of America’s Families investigated differences in insurance coverage, access, and health care utilization between Native Americans and non-Hispanic Caucasians. The researchers noted that Native Americans had lower rates of employer/other coverage and higher rates of public/state coverage. Sixteen percent of Native Americans reported IHS coverage only, and an additional 19% were uninsured and did not report the IHS as a source of coverage [48]. Native Americans have an uninsured rate almost three times that of non-Hispanic Caucasians and only about half of uninsured Native Americans reported having access to IHS care [48]. Compared to non-Hispanic Caucasians, Native Americans reported challenges accessing health care, dissatisfaction with the quality of health care received and communication issues with providers [48].

Knowledge is essential to recognizing the first signs of heart attack and stroke for timely and necessary care. We believe that pharmacists can play an important role in educating patients about the symptoms of heart attack and stroke because pharmacists are easily accessible HCPs [49]. Pharmacists have established positive roles in public health initiatives through providing education and interventions for a variety of disease states including hypertension, hyperlipidemia, and diabetes as well as smoking cessation [50–57]. In dispensing settings, pharmacists can provide valuable education and information regarding stroke and heart attack symptoms as nearly 90% of all Americans live within 5 miles of a community pharmacy [58]. Pharmacists are positioned as one of the most accessible HCPs, given that pharmacists see their patients between 1.5 to 10 times more frequently than primary care physicians see their patients [59]. Clinical pharmacists and other health care professionals who provide care for Native American patients are also in positions which allow them to provide education about stroke and heart attack symptomology when providing direct patient care. Moreover, working as part of a health care team constituted of physicians, advanced practice clinicians (nurse practitioners and physician assistants), dietitians, pharmacists and others can enhance coordination of care by providing essential medication and health education that may lead to better health outcomes as well as a subsequent reduction in health care costs.

### Limitations

Although BRFSS provides a national database with a robust sample weighted to reflect the demographics of the US population, there are a few possible limitations to this study. First, those who could not be reached by telephone could not respond to BRFSS survey as data is collected in a random-digit dial fashion. This could cause data to be skewed as persons of lower socioeconomic status might not have not been included in the survey due to poorer phone access. However, few US residents live in households without telephones and US cell phone numbers are now included in the BRFSS call list, which lessens this bias. Secondly, people may passively refuse to participate in surveys like the BRFSS by filtering their phone calls through caller identification and answering machines. However, survey work sponsored by the government is exempted from the Do Not Call Register and any potential call filtering cannot be controlled by the survey administrators. A third limitation is that the BRFSS survey does not use open-ended questions. This limits the respondents’ opportunities to fully explain their answers; but the survey questions are worded so that the answer choices cover a broad range of responses. Additionally, there is the possibility of recall bias by the survey participant as all of the survey answers are self-reported. Finally, the survey is administered in the English and Spanish languages. Participants who did not speak either languages where excluded and a proportion of Native Americans are likely to not speak either language.

## Conclusions

The analysis in this study identified characteristics of Native American adults who had low knowledge levels regarding heart attack and stroke symptoms. The knowledge deficits were greater for Native Americans than for other racial and ethnic groups in the US. These characteristics of Native American adults with knowledge deficits or disparities should guide educational initiatives and programs targeted at patients with low knowledge of heart attack and stroke symptoms scores. Interprofessional health care teams, including pharmacists, should be used to develop and implement health education interventions.

## Data Availability

Annual BRFSS data is publically available from the CDC at: https://www.cdc.gov/brfss/annual_data/annual_data.htm

## Abbreviations

AI: American Indians
AN: Alaskan Natives
BRFSS: Behavioral Risk Factor Surveillance System
CABG: Coronary Artery Bypass Graft
CVD: Cardiovascular disease
HCP: Health care provider
IHS: Indian Health Service
IRB: Institutional Review Board
MSA: Metropolitan Statistical Area
PCI: Percutaneous coronary intervention
US: United States
USVI: United States Virgin Islands
